# Семаглутид — эффективность в снижении веса и побочные эффекты при применении по данным исследований SUSTAIN, PIONEER, STEP

**DOI:** 10.14341/probl13197

**Published:** 2023-06-30

**Authors:** О. Р. Шабутдинова, А. Р. Даутов, А. А. Самков, А. В. Кононенко, А. Ф. Саргалиев, А. Р. Давлетшин, П. А. Андресова, К. Р. Зарбеева, Д. А. Торшхоева, У. А. Рахмонкулов, А. А. Афанасьев

**Affiliations:** Башкирский государственный медицинский университет; Башкирский государственный медицинский университет; Башкирский государственный медицинский университет; Башкирский государственный медицинский университет; Башкирский государственный медицинский университет; Башкирский государственный медицинский университет; Башкирский государственный медицинский университет; Башкирский государственный медицинский университет; Дагестанский государственный медицинский университет; Башкирский государственный медицинский университет; Самарский государственный медицинский университет

**Keywords:** ожирение, семаглутид, сахарный диабет, SUSTAIN, PIONEER, STEP, терапия ожирения

## Abstract

Избыточный вес и ожирение являются всемирно распространенной проблемой и диагностируются при значении индекса массы тела (ИМТ) в пределах 25,0–29,9 кг/ м2 и ≥30,0 кг/м2 соответственно. Пациенты с ожирением подвержены высокому риску развития сопутствующих заболеваний, таких как гипертоническая болезнь, сахарный диабет 2 типа (СД2), гиперлипидемия, инсульт и даже некоторые виды онкологических заболеваний. В Российской Федерации на 2016 г. доля лиц с избыточной массой тела составила 62,0%, с ожирением — 26,2%. Авторами был произведен электронный поиск в информационной базе данных PubMed. Были использованы два элемента поиска: «Semaglutide» и «Obesity». В поиск включались исследования, опубликованные с даты основания базы данных по август 2022 г. ­Поиск был ограничен только результатами клинических исследований. Авторами было получено 26 результатов, однако были рассмотрены только исследования SUSTAIN, PIONEER (Peptide Innovation for Early Diabetes Treatment) и STEP, поскольку они были оригинальными рандомизированными контролируемыми клиническими испытаниями, проведенными до утверждения семаглутида для терапии СД2 и ожирения.

## Abstract (en)

Overweight and obesity are a worldwide common problem and are diagnosed with a body mass index (BMI) value in the range of 25.0–29.9 kg/m2 and ≥30.0 kg/m2, respectively. Obese patients are at high risk of developing concomitant diseases, such as hypertension, type 2 diabetes mellitus (DM2), hyperlipidemia, stroke and even some types of cancer. In the Russian Federation in 2016, the proportion of overweight people was 62.0%, with obesity — 26.2%. The authors performed an electronic search in the PubMed information database. Two search elements were used: «Semaglutide» and «Obesity». The search included studies published from the date of foundation of the database to August 2022. The search was limited only to the results of clinical trials. The authors obtained 26 results, but only the studies of SUSTAIN, PIONEER (Peptide Innovation for Early Diabetes Treatment) and STEP were considered, since they were original, randomized, controlled clinical trials conducted before the approval of semaglutide for the treatment of DM2 and obesity.

## ВВЕДЕНИЕ

Избыточный вес и ожирение являются всемирно распространенной проблемой и диагностируются при значении индекса массы тела (ИМТ) в пределах 25,0–29,9 кг/м2 и ≥30,0 кг/м2 соответственно [1][2]. Пациенты с ожирением подвержены высокому риску развития сопутствующих заболеваний, таких как гипертоническая болезнь, сахарный диабет 2 типа (СД2), дислипидемия, инсульт и некоторые виды онкологических заболеваний [3][4]. Повышенный риск развития сердечно-сосудистых заболеваний является наиболее распространенной причиной смерти у пациентов с ожирением [5].

По данным Всемирной организации здравоохранения, в 2016 г. более 1,9 млрд взрослых (от 18 лет и старше) имели избыточную массу тела. Из них более 650 млн страдали ожирением. Распространенность ожирения среди мужчин составляла 11%, среди женщин — 15% [2]. По прогнозам, к 2030 г. 60% населения мира могут иметь избыточную массу тела или ожирение, если тенденции заболеваемости ожирением сохранятся [4]. В Российской Федерации на 2016 г. доля лиц с избыточной массой тела составила 62,0%, с ожирением — 26,2% [3].

Рекомендации по лечению избыточного веса и ожирения в Российской Федерации предусматривают изменение образа жизни посредством коррекции питания и расширения объема физических нагрузок в качестве первых шагов терапии [3]. Изменение образа жизни может снизить риск развития сердечно-сосудистых осложнений, но зачастую пациентам нелегко поддерживать необходимый вес [1]. Если никаких существенных изменений в результате коррекции образа жизни не происходит, добавление фармакотерапевтических средств может способствовать снижению веса [1].

В связи с большой распространенностью ожирения и особенностями системы оказания медицинской помощи рынок средств для снижения веса наиболее обширен именно в США. Управлением по пищевым и лекарственным продуктам США (FDA) в настоящее время одобрены четыре препарата для похудения, а именно фентермин-топирамат, орлистат, налтрексон-бупропион и агонист рецепторов глюкагоноподобного пептида-1 (АР ГПП-1) лираглутид [1]. Лоркасерин ранее получил одобрение FDA, но был отозван в начале 2020 г. из-за повышенного риска развития злокачественных новообразований [6]. Семаглутид является АР ГПП-1, который получил одобрение FDA в 2021 [7].

АР ГПП-1 неоднократно демонстрировали многообещающие результаты в снижении массы тела у пациентов с ожирением, страдающих диабетом и без него [3][4][8][9]. Они также эффективно улучшают гликемический контроль, стимулируя секрецию инсулина и ингибируя секрецию глюкагона без риска гипогликемии [1][10]. Хотя эффекты в отношении снижения веса хорошо известны, механизм, лежащий в их основе, все еще остается спорным. Большинство исследований, посвященных изучению основных механизмов влияния ГПП-1 на аппетит и снижение веса, было сосредоточено на лираглутиде [1]. Наиболее известные механизмы связаны с воздействием на центральную и периферическую нервную систему посредством специфической активации рецепторов ГПП-1 в гипоталамусе и заднем мозге или посредством косвенной активации через блуждающий нерв, что приводит к усилению сигналов насыщения и ослаблению сигналов голода [3]. Сигналы от ядра солитарного тракта в продолговатом мозге к вентральной тегментальной области и прилежащему ядру могут вовлекать ГПП-1 во влияние на пищевые мотивационные реакции, а также снижать общую вкусовую привлекательность [3]. Известно также, что АР ГПП-1 задерживают опорожнение желудка, но влияние этого эффекта на общее снижение веса пациентов, по-видимому, минимально [3]. В целом вышеуказанные механизмы влияют на потребление энергии, а не на скорость метаболизма в состоянии покоя [8].

В исследованиях также изучались побочные эффекты со стороны желудочно-кишечного тракта (ЖКТ), а именно тошнота и рвота, которые наиболее часто встречались у пациентов. На основании данных программы клинических исследований SUSTAIN (Semaglutide Unabated Sustainability in Treatment of Type 2 Diabetes) отмечено, что побочные эффекты в виде тошноты и рвоты не были связаны со снижением веса пациентов [8]. В вышеупомянутом исследовании также оценивались основные механизмы снижения веса у пациентов, принимавших семаглутид (Оземпик), относительно новый АР ГПП-1, который в настоящее время представлен на рынке только в качестве противодиабетического препарата как в форме инъекций, так и в форме таблеток для приема внутрь [8]. STEP (The Semaglutide Treatment Effect in People with Obesity) — это программа клинических испытаний III фазы, направленная на одобрение семаглутида в качестве средства для снижения веса у пациентов с ожирением. Она включала пять исследований, в основном направленных на сравнение семаглутида для подкожного введения (2,4 мг 1 раз в неделю) с плацебо [11–15]. В этой исследовательской программе семаглутид не сравнивался с другими препаратами для терапии ожирения, представленными в настоящее время на рынке. Результаты показали, что семаглутид в сочетании с коррекцией образа жизни приводит к клинически значимому снижению веса у пациентов с ожирением по сравнению с плацебо [11–15].

Семаглутид продемонстрировал не только улучшение показателей массы тела, но и снижение смертности от сердечно-сосудистых осложнений у пациентов с СД2 [16].

## МАТЕРИАЛЫ И МЕТОДЫ

Авторами был произведен электронный поиск в информационной базе данных PubMed. Были использованы два элемента поиска: «Semaglutide» и «Obesity». В поиск включались исследования, опубликованные с даты основания базы данных по август 2022 г. Поиск был ограничен только результатами клинических исследований. Авторами было получено 26 результатов, однако были рассмотрены только исследования SUSTAIN, PIONEER (Peptide Innovation for Early Diabetes Treatment) и STEP, поскольку они были оригинальными рандомизированными контролируемыми клиническими испытаниями, проведенными до утверждения семаглутида для терапии СД2 и ожирения.

Три из 13 исследований, включавших программу SUSTAIN, были исключены из настоящего обзора ввиду избыточности сравниваемых препаратов [17–19].

## Исследования SUSTAIN

Недавняя программа клинических исследований SUSTAIN изучала безопасность и эффективность семаглутида для подкожного введения [10]. Добавление жирной двухкислотной цепи в положение 26 улучшает связывание препарата с альбумином, а замена аланина на α-аминоизомасляную кислоту в положении 8 делает молекулу менее восприимчивой к деградации дипептидилпептидазой-4. Эти модификации привели к увеличению периода полураспада молекулы, что позволяет вводить семаглутид подкожно 1 раз в неделю [10].

На основании анализа 10 исследований (табл. 1) было обнаружено, что семаглутид эффективнее снижал уровень гликированного гемоглобина (HbA1c), а также массу тела испытуемых по сравнению с плацебо [16][20–22] и с несколькими противодиабетическими препаратами, включая инсулин гларгин [23], ситаглиптин [8], а также эксенатид с пролонгированным высвобождением (ПВ) [10], дулаглутид [24], канаглифлозин [25] и лираглутид [26]. Кроме того, преимущества АР ГПП-1 в снижении веса и улучшении гликемического контроля были продемонстрированы в предыдущем клиническом исследовании, в котором изучались безопасность и эффективность других препаратов этого класса [4]. Однако, имея в виду это сходство, важно отметить, что снижение массы тела пациентов, принимавших семаглутид, по сравнению с аналогами, было эффективнее, чем в других программах клинических исследований АР ГПП-1 [4]. В исследованиях SUSTAIN семаглутид демонстрировал снижение массы тела по сравнению с другими АР ГПП-1 (табл. 1). Сравнение других АР ГПП-1 длительного действия с семаглутидом, а именно эксенатида ПВ [10] и дулаглутида [24], показало преимущества семаглутида в общем снижении веса, а также в количестве пациентов, достигших потери веса ≥5% [4].

Исследование SUSTAIN 10 показало, что семаглутид также имеет преимущества перед лираглутидом в общем снижении массы тела и количестве пациентов, достигших потери веса ≥5% и ≥10% (табл. 1) [23]. Следует отметить, что подкожное введение семаглутида требуется только 1 раз в неделю, тогда как лираглутид необходимо вводить ежедневно. Это может обеспечить более высокий комплаенс пациентов с ожирением. Фактически в большинстве исследований SUSTAIN пациенты, получавшие семаглутид, сообщали о более высокой общей удовлетворенности лечением по сравнению с другими группами [27].

**Table:** Таблица 1. Краткое изложение результатов SUSTAINTable 1. Summary of SUSTAIN results * — Р<0,05, что указывает на превосходство семаглутида по сравнению с соответствующим препаратом.** — отдельно или в сочетании друг с другом.ПВ — пролонгированное высвобождение; НЗ — не зарегистрировано; ПК — подкожный; SGLT-2 — натрий-глюкозный транспортер 2 типа.

SUSTAIN 1 (n=388) [13]	Диета и физические упражнения	ПК семаглутид 0,5 мг	–3,73*	37*
ПК семаглутид 1,0 мг	–4,53*	45*
Плацебо	–0,98	7
SUSTAIN 2 (n=1231) [8]	Метформин, пиоглитазон, росиглитазон	ПК семаглутид 0,5 мг	–4,3*	46*
ПК семаглутид 1,0 мг	–6,1*	62*
Ситаглиптин 100 мг	–1,9	18
SUSTAIN 3 (n=809) [9]	Метформин, сульфонилмочевина, тиазолидиндион	ПК семаглутид 1,0 мг	–5,6*	52*
Эксенатид пролонгированного высвобождения (ПВ) 2 мг	–1,9	17
SUSTAIN 4 (n=1089) [15]	Метформин, сульфонилмочевина	ПК семаглутид 0,5 мг	–3.47*	37*
ПК семаглутид 1,0 мг	–5,17*	51*
Инсулин гларгин	-1,15	5
SUSTAIN 5 (n=397) [12]	Метформин, базальный инсулин	ПК семаглутид 0,5 мг	–3,7*	42*
ПК семаглутид 1,0 мг	–6,4*	66*
Плацебо	–1,4	11
SUSTAIN 6 (n=3297) [11]	0–2 антигипергликемических средства	ПК семаглутид 0,5 мг	–3,6*	НЗ
ПК семаглутид 1,0 мг	–4,9*	НЗ
Плацебо 1,0 мг	–0,7	НЗ
Плацебо 0,5 мг	–0,5	НЗ
SUSTAIN 7 (n=1201) [16]	Метформин	ПК семаглутид 0,5 мг	–4,6*	44*
Дулаглутид 0,75 мг	–2,3	23
ПК семаглутид 1,0 мг	–2,3*	63*
Дулаглутид 1,5 мг	–1,1	30
SUSTAIN 8 (n=788) [17]	Метформин	ПК семаглутид 1,0 мг	–5,3*	53
Канаглифлозин 300 мг	–4,2	47
SUSTAIN 9 (n=302) [14]	Метформин, сульфонилмочевина, ингибитор SGLT-2	ПК семаглутид 1,0 мг	–4,7*	50*
Плацебо	–0,9	8
SUSTAIN 10 (n=577) [18]	Метформин, ингибитор SGLT-2	ПК семаглутид 1,0 мг	–5,8*	56*
Лираглутид 1,2 мг	–1,9	18

Наряду с повышением удовлетворенности пациентов, одобрение семаглутида в качестве лекарства для коррекции массы тела может привести к расширению возможностей для изучения многих основных механизмов снижения веса препаратов группы АР ГПП-1. В настоящее время большинство исследований на животных и людях, изучающих эти механизмы, было проведено с использованием лираглутида [1][3]. Наконец, АР ГПП-1, такие как лираглутид и семаглутид, обладают кардиопротекторными свойствами [16], которые весьма актуальны, учитывая, что сердечно-сосудистые факторы риска обычно присутствуют у пациентов с ожирением [3]. Побочные эффекты со стороны желудочно-кишечного тракта, включая тошноту и рвоту, были наиболее распространенными и приводили к прекращению приема семаглутида у 8% пациентов (табл. 2). На рисунках 1–5 представлена частота побочных эффектов со стороны ЖКТ по степени тяжести.

**Table:** Таблица 2. Краткое изложение результатов SUSTAIN в области безопасностиTable 2. Summary of SUSTAIN Security Results * — 2 случая тяжелой гипогликемии в исследовании SUSTAIN 3 были в группе ПК семаглутида в дозе 1,0 мг.ПЭ — побочные эффекты; ПВ — пролонгированное высвобождение; ЖКТ — желудочно-кишечный тракт; НЗ — не зарегистрировано; ПК — подкожный.

Исследование (n)	Доза и препараты сравнения	Продолжительность терапии, нед	ПЭ, %	Прекращение лечения из-за ПЭ, %	Прекращение приема препарата из-за ПЭ со стороны ЖКТ, %	Тяжелая гипогликемия, %
SUSTAIN 1 (n=388) [13]	ПК семаглутид 0,5 мг	30	64	6	4	Нет
ПК семаглутид 1,0 мг	56	5	3
Плацебо	53	2	<1
SUSTAIN 2 (n=1231) [8]	ПК семаглутид 0,5 мг	56	75	8	7	НЗ
ПК семаглутид 1,0 мг	71	10	8
Ситаглиптин 100 мг	72	3	3
SUSTAIN 3 (n=809) [9]	ПК семаглутид 1,0 мг	56	75	9	НЗ	2 события*
Эксенатид ПВ 2 мг	76	7
SUSTAIN 4 (n=1089) [15]	ПК семаглутид 0,5 мг	30 нед	70	6	3	НЗ
ПК семаглутид 1,0 мг	73	7	5
Инсулин гларгин	65	1	0
SUSTAIN 5 (n=397) [12]	ПК семаглутид 0,5 мг	30	69	5	НЗ	8
ПК семаглутид 1,0 мг	64	6	11
Плацебо	58	<1	5
SUSTAIN 6 (n=3297) [11]	ПК семаглутид 0,5 мг	104	90	12	НЗ	23
ПК семаглутид 1,0 мг	89	15	22
Плацебо	91	6	22
Плацебо	89	8	21
SUSTAIN 7 (n=1201) [16]	ПК семаглутид 0,5 мг	40	68	8	5	3
Дулаглутид 0,75 мг	62	5	2	1
ПК семаглутид 1,0 мг	69	10	6	2
Дулаглутид 1,5 мг	74	7	5	2
SUSTAIN 8 (n=788) [17]	ПК семаглутид 1,0 мг	52	76	10	7	2
Канаглифлозин 300 мг	72	5	1	1
SUSTAIN 9 (n=302) [14]	ПК семаглутид 1,0 мг	30	69	9	7	11
Плацебо	60	2	0	2
SUSTAIN 10 (n=577) [18]	ПК семаглутид 1,0 мг	30	71	11	7	НЗ
Лираглутид 1,2 мг	66	7	4

**Figure:**
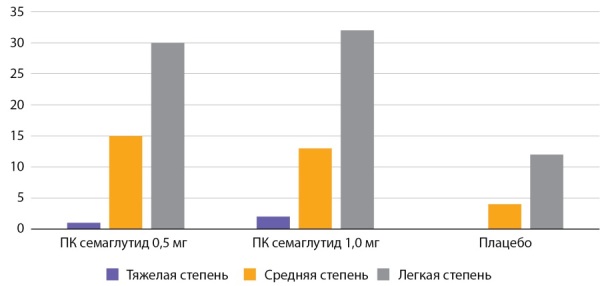
Рисунок 1. Частота побочных эффектов со стороны ЖКТ по данным исследования SUSTAIN 1 (n=388) (%).Figure 1. The frequency of side effects from the gastrointestinal tract according to the study SUSTAIN 1 (n=388) (%).

**Figure:**
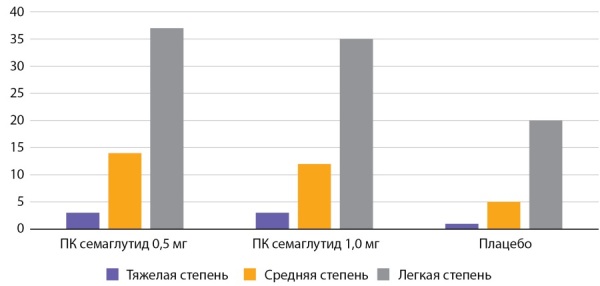
Рисунок 2. Частота побочных эффектов со стороны ЖКТ по данным исследования SUSTAIN 2 (n=1231) (%).Figure 2. The frequency of side effects from the gastrointestinal tract according to the study SUSTAIN 2 (n=1231) (%).

**Figure:**
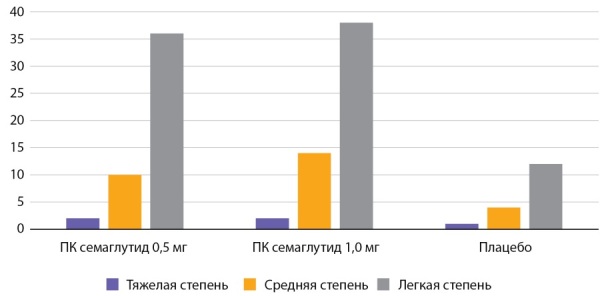
Рисунок 3. Частота побочных эффектов со стороны ЖКТ по данным исследования SUSTAIN 4 (n=1089) (%).Figure 3. The frequency of side effects from the gastrointestinal tract according to the study SUSTAIN 4 (n=1089) (%)

**Figure:**
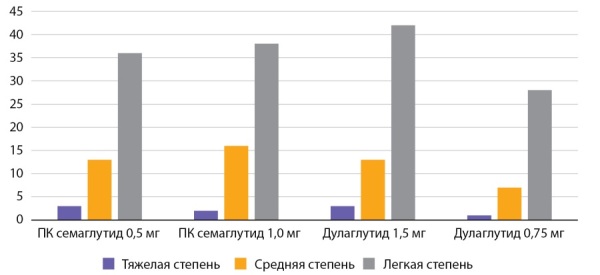
Рисунок 4. Частота побочных эффектов со стороны ЖКТ по данным исследования SUSTAIN 7 (n=1201) (%).Figure 4. The frequency of side effects from the gastrointestinal tract according to the study SUSTAIN 7 (n=1201) (%).

**Figure:**
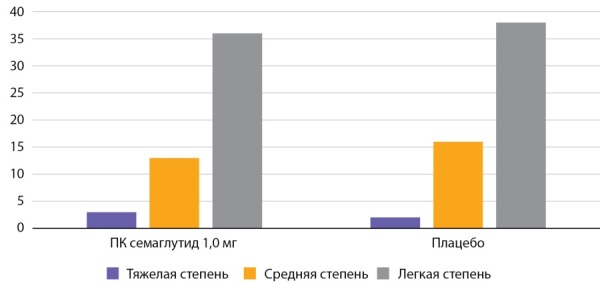
Рисунок 5. Частота побочных эффектов со стороны ЖКТ по данным исследования SUSTAIN 9 (n=302) (%).Figure 5. The frequency of side effects from the gastrointestinal tract according to the study SUSTAIN 9 (n=302) (%)

## Исследования PIONEER

Наряду с доказательствами, полученными в ходе программы клинических исследований SUSTAIN, была проведена вторая программа для оценки безопасности и эффективности перорального семаглутида (Ребелсас) в качестве противодиабетического препарата. Программа клинических исследований PIONEER (Peptide Innovation for Early Diabetes Treatment) была направлена на получение данных об эффективности гликемического контроля и снижения массы тела у пациентов с СД2 во время лечения пероральным семаглутидом в сравнении с другими противодиабетическими препаратами. Семаглутид в настоящее время является единственным пероральным препаратом в своей группе, все остальные АР ГПП-1 вводятся подкожно [28][29]. Пероральный семаглутид модифицируется добавлением усилителя абсорбции N-(8-[ 2-гидроксибензоил]амино) каприлата натрия [28][29]. Учитывая низкую биодоступность, в таблетках содержится увеличенная доза действующего вещества; результаты программы клинических исследований PIONEER показывают, что пероральный семаглутид в дозах 7 и 14 мг показал более эффективное снижение уровня HbA1c по сравнению с ситаглиптином, однако семаглутид в дозе 3 мг не показал клинически значимого эффекта [30].

На протяжении клинических испытаний PIONEER (табл. 3) неоднократно наблюдались преимущества в виде более выраженного снижения HbA1c и массы тела при пероральном приеме семаглутида по сравнению с плацебо [31–35]. Преимущества перорального семаглутида также наблюдались при сравнении с другими пероральными противодиабетическими препаратами, такими как ситаглиптин [30][36] и эмпаглифлозин [26], которые выражались в более выраженном снижении HbA1c и массы тела. Кроме того, преимущества перорального семаглутида были также обнаружены при сравнении с препаратами того же класса лекарств (дулаглутид [37] и лираглутид [33][34]). Подобно клиническим испытаниям SUSTAIN, исследования PIONEER показали улучшение гликемического контроля и снижение веса при приеме семаглутида внутрь в качестве дополнения к монотерапии метформином.

В исследованиях PIONEER пероральный семаглутид превзошел другие АР ГПП-1 — дулаглутид и лираглутид — в отношении снижения веса [33–35] (табл. 3). Также наблюдались другие положительные эффекты, такие как уменьшение окружности талии и ИМТ пациентов. У большего числа пациентов, получавших семаглутид перорально, снижение веса составило не менее 5% и не менее 10% по сравнению с теми пациентами, которые получали препараты сравнения [33][36]. По сравнению с лираглутидом пероральный прием семаглутида показал более выраженный эффект в отношении снижения массы тела [33], который, как было обнаружено позже, является дозозависимым [34]. Пациенты, принимающие семаглутид перорально, имеют более высокую вероятность снижения веса на ≥5% и на ≥10% по сравнению с лираглутидом (табл. 3) [33]. Максимальное снижение массы тела было установлено к 52-й неделе [28][36], однако о значительном снижении веса сообщалось уже через 26 нед приема препарата [30][33][35].

**Table:** Таблица 3. Краткое изложение результатов PIONEERTable 3. Summary of PIONEER Results * — Р<0,05, что указывает на превосходство семаглутида по сравнению с соответствующим препаратом.** — отдельно или в сочетании друг с другом.НЗ — не зарегистрировано; SGLT-2 — натрий-глюкозный транспортер 2 типа.

Исследование (n)	Фоновая терапия**	Доза и препараты сравнения	Изменение массы тела по сравнению с исходным уровнем, кг	Пациенты с потерей веса >5%, %
PIONEER 1(n=703) [23]	Диета и физические упражнения	Пероральный семаглутид 3 мг	–1,5	20
Пероральный семаглутид 7 мг	–2,3	27
Пероральный семаглутид 14 мг	–3,7*	40*
Плацебо	–1,4	15
PIONEER 2 (n=822) [20]	Метформин	Пероральный семаглутид 14 мг	–3,8	41
Эмпаглифлозин 25 мг	–3,7	36
PIONEER 3 (n=1864) [22]	Метформин, сульфонилмочевина	Пероральный семаглутид 3 мг	–1,2	13
Пероральный семаглутид 7 мг	–2,2*	19*
Пероральный семаглутид 14 мг	–3,1*	30*
Ситаглиптин 100 мг	–0,6	10
PIONEER 4(n=711) [25]	Метформин, ингибитор SGLT-2	Пероральный семаглутид 14 мг	–4,4*	44*
Лираглутид 1,8 мг	–3,1	28
Плацебо	–0,5	8
PIONEER 5(n= 324) [24]	Метформин, базальный инсулин, сульфонилмочевина	Пероральный семаглутид 14 мг	–3,4*	36*
Плацебо	–0,9	10
PIONEER 6 (n=3183) [30]	Никаких исключений в зависимости от режима	Пероральный семаглутид 14 мг	–4,2	НЗ
Плацебо	–0,8	НЗ
PIONEER 7(n=504) [28]	1–2 антигипергликемических средства	Пероральный семаглутид (гибкая доза 3, 7 или 14 мг)	–2,6*	27*
Ситаглиптин 100 мг	–0,7	12
PIONEER 8(n=731) [27]	Метформин, инсулин (базальный, базально-болюсный или предварительно смешанный)	Пероральный семаглутид 3 мг	–1,4*	13*
Пероральный семаглутид 7 мг	–2,4*	31*
Пероральный семаглутид 14 мг	–3,7*	39*
Плацебо	–0,4	3
PIONEER 9(n=243) [26]	Диета и физические упражнения или 1 антигипергликемическое средство	Пероральный семаглутид 3 мг	–0,6	4
Пероральный семаглутид 7 мг	–1,1*	10
Пероральный семаглутид 14 мг	–2,4*	34*
Лираглутид 0,9 мг	0,0	0
Плацебо	–1,1	10
PIONEER 10 (n= 458) [29]	1 антигипергликемическое средство	Пероральный семаглутид 3 мг	–0,2	5
Пероральный семаглутид 7 мг	–1,0*	18*
Пероральный семаглутид 14 мг	–2,2*	31*
Дулаглутид 0,75 мг	–0,3	6

Что касается безопасности, сообщалось, что пероральный прием семаглутида не уступает плацебо в снижении частоты серьезных нежелательных сердечно-сосудистых событий [28][38]. Безопасность перорального приема семаглутида у пациентов с умеренной почечной недостаточностью (скорость клубочковой фильтрации от 30 до 59%) также была подтверждена в исследовании PIONEER 5 [32], при этом на протяжении всего исследования не наблюдалось каких-либо существенных изменений со стороны почек. Как и в исследованиях SUSTAIN, пероральный прием семаглутида не выявил повышенного риска развития гипогликемии [30]. Наиболее часто регистрируемыми были побочные эффекты со стороны ЖКТ (табл. 4), а именно легкая или умеренная тошнота и рвота [30–35][37]. Наконец, было отмечено значительное увеличение активности липазы [31].

**Table:** Таблица 4. Краткое изложение результатов PIONEER в области безопасностиTable 4. Summary of PIONEER Safety Results * Текущее исследование.ПЭ — побочные эффекты; ЖКТ — желудочно-кишечный тракт; НЗ — не зарегистрировано.

Исследование (количество участников)	Доза и препаратысравнения	Продолжительность терапии, нед	ПЭ, %	Прекращение лечения из-за ПЭ, %	Прекращение приема препарата из-за ПЭ со стороны ЖКТ, %	Тяжелая гипогликемия, %
PIONEER 1 (n=703) [23]	Пероральный семаглутид 3 мг	26	58	2	2	3
Пероральный семаглутид 7 мг		53	4	2	1
Пероральный семаглутид 14 мг		57	7	5	1
Плацебо		56	2	1	1
PIONEER 2 (n=822) [20]	Пероральный семаглутид 14 мг	52	70	11	8	2
Эмпаглифлозин 25 мг		69	4	1	2
PIONEER 3 (n=1864) [22]	Пероральный семаглутид 3 мг	78	79	6	2	5
Пероральный семаглутид 7 мг		78	6	3	5
Пероральный семаглутид 14 мг		80	12	7	8
Ситаглиптин 100 мг		83	5	3	8
PIONEER 4 (n=711) [25]	Пероральный семаглутид 14 мг	52	80	11	8	1
Лираглутид 1,8 мг		74	9	6	2
Плацебо		67	4	2	2
PIONEER 5 (n=324) [24]	Пероральный семаглутид 14 мг	26	74	15	12	6
Плацебо		65	5	2	2
PIONEER 6 (n=3183) [30]	Пероральный семаглутид 14 мг	*	НЗ	12	7	НЗ
Плацебо			7	2	НЗ
PIONEER 7 (n=504) [28]	Пероральный семаглутид (гибкая доза 3, 7 или 14 мг)	52	78	9	6	6
Ситаглиптин 100 мг		69	3	1	6
PIONEER 8 (n=731) [27]	Пероральный семаглутид 3 мг	52	74	7	5	28
Пероральный семаглутид 7 мг		78	9	7	26
Пероральный семаглутид 14 мг		83	13	10	27
Плацебо		76	3	1	29
PIONEER 9(n=243) [26]	Пероральный семаглутид 3 мг	52	76	2	2	0
Пероральный семаглутид 7 мг		76	4	2	0
Пероральный семаглутид 14 мг		71	0	4	0
Лираглутид 0,9 мг		67	0	0	4
Плацебо		80	0	0	0
PIONEER 10(n=458) [29]	Пероральный семаглутид 3 мг	52	77	3	31	2
Пероральный семаглутид 7 мг		80	6	39	2
Пероральный семаглутид 14 мг		85	6	54	3
Дулаглутид 0,75 мг		82	3	40	0

## Исследования STEP

Семаглутид был исследован не только в программе лечения СД, где снижение веса являлось побочным, хотя и желательным результатом лечения, но и непосредственно для лечения ожирения у людей без диабета. Самой последней программой клинических исследований по изучению эффективности подкожного введения семаглутида была программа STEP. Основной ее целью была оценка эффективности семаглутида в качестве средства для снижения массы тела. Участники исследований отбирались на основании ИМТ, пациенты с СД2 не включались в исследование [11][13–15], за исключением STEP 2 [12]. Экспериментальная доза, определенная в ходе исследования, составляла 2,4 мг и вводилась подкожно 1 раз в неделю. Программа была завершена в марте 2021 г. и включала в общей сложности пять исследований (табл. 5). Результаты STEP 1–4 показали, что семаглутид более эффективно снижал массу тела по сравнению с плацебо [11–14]. В STEP 2 проводилось сравнение 2,4 мг и 1,0 мг семаглутида, результаты показали, что прием 2,4 мг семаглутида приводит к более значительному снижению веса, чем 1,0 мг [12]. В STEP 4 изучались последствия прекращения лечения семаглутидом и было обнаружено, что у пациентов, которые начали принимать плацебо после 20 нед терапии экспериментальной дозой семаглутида, наблюдалось увеличение веса примерно на 6 кг [14] (табл. 5). Было обнаружено, что профиль безопасности 2,4 мг семаглутида аналогичен профилю безопасности 1,0 мг семаглутида для подкожного введения и перорального приема, при этом побочные эффекты со стороны ЖКТ легкой и средней степени тяжести были основной жалобой среди участников исследования (табл. 6). О случаях гипогликемии сообщалось нечасто, что является обнадеживающим фактом для назначения семаглутида пациентам без СД2. Исследование STEP 5 проходило на протяжении двух лет. В нем оценивались эффективность и безопасность еженедельного подкожного введения семаглутида в дозе 2,4 мг по сравнению с плацебо для длительной терапии взрослых с ожирением или избыточным весом, по крайней мере, с одним сопутствующим заболеванием без СД. По результатам исследования больше участников в группе семаглутида, чем в группе плацебо, достигли снижения веса на ≥5% по сравнению с исходным уровнем на 104-й неделе (77,1% против 34,4%; Р<0,0001). О нежелательных явлениях со стороны ЖКТ, в основном легкой и умеренной степени, при применении семаглутида сообщалось чаще, чем при применении плацебо (82,2% против 53,9%). Таким образом, у взрослых с избыточным весом или ожирением лечение семаглутидом приводило к существенному и устойчивому снижению веса в течение 104 нед по сравнению с плацебо [15].

**Table:** Таблица 5. Краткое изложение результатов STEPTable 5. Summary of STEP results ПК — подкожный; НЗ — не зарегистрировано.

STEP 1(n=1961) [11]	ПК семаглутид 2,4 мг	−14,9	86,4, 69,1, 50,5	−15,3
Плацебо	−2,4	31,5, 12,0, 4,9	−2,6
STEP 2(n=1210) [12]	ПК семаглутид 2,4 мг	−9,6	68,8, 45,6, 25,8	−9,7
ПК семаглутид 1,0 мг	−7,0	57,1, 28,7, 13,7	−2,5
Плацебо	−3,4	28,5, 8,2, 3,2	−1,3
STEP 3(n=611) [13]	ПК семаглутид 2,4 мг	−16	86,6, 75,3, 55,8	−16,8
Плацебо (+интенсивная поведенческая терапия)	−5,7	47,6, 27, 13,2	−6,2
STEP 4(n=902) [14]	ПК семаглутид (всего 68 нед)	−7,9	НЗ	−7,1
ПК семаглутид (20 нед), затем плацебо (48 нед)	+6,9	НЗ	+6,1
STEP 5(n=304) [15]	ПК семаглутид 2,4 мг	-15,6	77,1, 61,8, 52,1	-16,1
Плацебо	-3,0	34,4, 13,3, 7	-3,2

**Table:** Таблица 6. Краткое изложение результатов STEP в области безопасностиTable 6. Summary of STEP Security Results ПЭ — побочные эффекты; ЖКТ — желудочно-кишечный тракт; ПК — подкожный.

Исследование (количество участников)	Доза и препараты сравнения	Продолжительность терапии, нед	ПЭ, %	Прекращение лечения из-за ПЭ, %	Прекращение приема препарата из-за ПЭ со стороны ЖКТ, %	Тяжелая гипогликемия, %
STEP 1(n=1961) [11]	ПК семаглутид 2,4 мг	68	86,7	74,2	4,5	0,6
Плацебо	86,4	47,9	0,8	0,8
STEP 2(n=1210)[12]	ПК семаглутид 2,4 мг	68	97,6	63,5	4,2	5,7
ПК семаглутид 1,0 мг	81,8	57,5	3,5	5.5
Плацебо	76,9	34,3	1,0	3,0
STEP 3(n=611)[13]	ПК семаглутид 2,4 мг	68	95,8	82,8	3,4	0,5
Плацебо (+интенсивная поведенческая терапия)	96,1	63,2	0	0
STEP 4(n=902)[14]	ПК семаглутид	68	81,3	49,1	2,4	0,6
Плацебо	75	26,1	2,2	1,1
STEP 5(n=304)[15]	ПК семаглутид 2,4 мг	104	96,1	5,9	3,9	0
Плацебо	89,5	4,6	0,7	0,7

Растущий объем данных программы клинических испытаний STEP продемонстрировал эффективность и переносимость подкожного введения семаглутида в дозе 2,4 мг 1 раз в неделю у лиц с избыточной массой тела или ожирением. Во всех исследованиях STEP подкожное введение семаглутида в дозе 2,4 мг 1 раз в неделю неизменно приводило к снижению веса в среднем на 14,9–17,4% у участников без СД и улучшению кардиометаболических факторов риска, физических функций и качества жизни. В процессе STEP 1–5 не проводилось прямого сравнения 2,4 мг семаглутида с другими одобренными FDA препаратами для снижения веса. Это было бы плодотворным направлением для будущих исследований фармакотерапевтических средств, направленных на снижение массы тела.

## Демографические данные исследований SUSTAIN, PIONEER и STEP

Исследования SUSTAIN 1–10, PIONEER и STEP являются рандомизированными контролируемыми многоцентровыми и многонациональными, за исключением PIONEER 9 и 10, проводимых в Японии.

Около 60–93% населения были белыми в исследованиях SUSTAIN 1–10, STEP и PIONEER 1–8 и около 2–10% были чернокожими или азиатами. Женщины составляли 40–50% в исследованиях SUSTAIN 1–10 и PIONEER 1–8, 20–30% в PIONEER 9 и 10, однако в исследованиях STEP большинство исследуемой популяции составляли женщины, на долю которых приходилось около 50–81%.

В связи с преобладанием европеоидной расы среди пациентов полученные данные можно достаточно надежно экстраполировать и на российских пациентов.

## Сравнение и обобщение фактических данных по семаглутиду

Преимущества семаглутида в снижении массы тела по сравнению с другими противодиабетическими препаратами были подробно описаны в вышеуказанных клинических исследованиях. В клиническом исследовании STEP была подтверждена эффективность еженедельного приема семаглутида в дозе 2,4 мг по сравнению с плацебо в отношении снижения массы тела пациентов с ожирением и без СД2 [11][13][14].

В данном разделе мы сравнили результаты, полученные для семаглутида, в зависимости от способа введения — пероральный прием или подкожная инъекция — на основании результатов исследований PIONEER и SUSTAIN (табл. 1 и 3). В исследованиях PIONEER было обнаружено, что прием перорального семаглутида в дозе 14 мг приводил к снижению веса на 2,3 кг, в то время как прием 0,5 и 1,0 мг семаглутида для подкожного введения приводил к снижению веса на 3,73 и 4,53 кг соответственно [21][31]. Аналогичным образом, по сравнению с ситаглиптином как пероральный, так и подкожный семаглутид показали преимущества в снижении массы тела; однако при косвенном сравнении результатов (табл. 1) мы обнаружили, что подкожное введение семаглутида приводило к большему снижению массы тела, чем пероральное [9][30][36]. Эта тенденция снова прослеживается при сравнении SUSTAIN 8 и PIONEER [25][28]. Однако нами не проводился статистический анализ с использованием исходных необработанных данных. Поэтому эти данные не следует рассматривать как окончательный вывод о превосходстве одного способа введения семаглутида над другим. Насколько нам известно, ни в одном из предыдущих исследований не сравнивались различия в эффективности перорального и подкожного введения семаглутида, что должно быть рассмотрено в будущих исследованиях. В программе STEP сравнивали прием 1,0 и 2,4 мг семаглутида для подкожного введения, по результатам исследований был выявлен дозозависимый эффект в отношении снижения массы тела [12].

Как пероральный семаглутид, так и подкожный вызывали сходные побочные эффекты (табл. 2 и 4), и наиболее часто отмечались желудочно-кишечные расстройства (в основном легкие или умеренные тошнота и рвота). Прекращение лечения из-за желудочно-кишечных осложнений варьировалось от 4,9 до 12% в исследованиях PIONEER [32][35] и от 3 до 9,4% в исследованиях SUSTAIN [10][23]. В обоих исследованиях также наблюдалось повышение уровня панкреатической липазы [10][21][23]. Частота эпизодов гипогликемии была низкой как при приеме перорального семаглутида [30], так и при введении семаглутида подкожно [23][39].

Единственным очевидным различием между пероральным семаглутидом и подкожным, помимо незначительных различий в их эффективности для снижения веса, является способ введения. В недавнем исследовании большинство пациентов сообщили о предпочтении перорального приема лекарства, что объясняется простотой введения и отсутствием болевых ощущений [40]. Исходя из этих результатов, большинство пациентов, возможно, предпочли бы принимать семаглутид перорально, а не путем еженедельной подкожной инъекции. В настоящее время FDA одобрило семаглутид (2,4 мг для подкожного введения) в качестве лекарственного средства для терапии ожирения [7]. В будущем важно изучить пероральный семаглутид в качестве средства для снижения веса, учитывая отсутствие различий в эффективности между пероральным и подкожным семаглутидом и возможное предпочтение перорального препарата в популяции пациентов.

Подкожная инъекция семаглутида представляет собой предварительно заполненный шприц-ручку с иглой диаметром всего 4 мм и калибром 32 мм, что делает ее удобной для пациента и простой в использовании.

## Семаглутид по сравнению с другими препаратами для снижения массы тела

В предыдущих разделах настоящего обзора мы обсудили эффективность семаглутида для снижения массы тела, используя опубликованные данные, сравнивающие семаглутид с плацебо или другими противодиабетическими препаратами, которые, как известно, вызывают снижение веса. Чтобы адекватно использовать потенциал семаглутида против ожирения, важно учитывать, достигается ли снижение массы тела по крайней мере на 5%, что рассматривается как клинически значимый параметр снижения веса [41]. Поэтому мы сравним результаты, наблюдаемые у семаглутида, с таковыми других препаратов для терапии ожирения (без противодиабетических свойств), а именно фентермина/топирамата, орлистата и налтрексона/бупропиона. Мы не включили лоркасерин (Белвик, Белвик ПВ), поскольку FDA отозвало его одобрение из-за повышенного риска развития злокачественных новообразований, о котором сообщалось в недавнем клиническом исследовании [6].

Что касается данных об эффективности, метаанализ 28 рандомизированных плацебо-контролируемых клинических исследований показал, что все 4 препарата против ожирения соответствуют порогу снижения веса FDA — не менее 5%. Фентермин/топирамат был наиболее эффективным препаратом, за которым следовал лираглутид, поскольку примерно 75 и 63% пациентов достигли этой цели соответственно [42]. Значительное число участников в каждом исследовании добились снижения веса по крайней мере на 10% по сравнению с плацебо, при этом фентермин/топирамат и лираглутид показали наилучшие результаты [42].

Что касается этой конечной точки, клинические исследования PIONEER, SUSTAIN и STEP (табл. 1 и 3) показали аналогичные эффекты снижения веса у участников, принимавших семаглутид [11][20–22][31][38]. На сегодняшний день отсутствуют исследования, сравнивающие влияние семаглутида и фентермина/топирамата на достижение 5% потери веса. Сравнивая результаты, представленные в обоих исследованиях, мы видим, что в первичном плацебо-контролируемом исследовании программы PIONEER и SUSTAIN 1 около 40% участников, принимавших семаглутид, добились снижения веса на 5% и более [21][31]. Таким образом, эти результаты могут свидетельствовать о том, что семаглутид превосходит фентермин/топирамат и лираглутид в качестве препаратов против ожирения. Тем не менее исследования этих двух препаратов для снижения массы тела также включали другие мероприятия по снижению веса, такие как программа гипокалорийной диеты [43][44] или консультации по диете и физическим упражнениям/изменению образа жизни [32][45][46]. Исследования III фазы программ SUSTAIN и PIONEER не включали подобные мероприятия. Исследование First STEP включало изменения образа жизни в качестве части своих экспериментальных требований и показало, что 86,5% участников, принимавших семаглутид, добились потери веса не менее чем на 5% [11]. Поэтому разумно предположить, что семаглутид в сочетании с другими мероприятиями по снижению веса будет соответствовать или превосходить достижения лираглутида или даже фентермина/топирамата. Необходимо проведение дальнейших исследований, посвященных этим сравнениям.

Следует также учитывать существенные различия в безопасности между этими препаратами. Как указывалось выше, семаглутид, как и другие АР ГПП-1, вызывает побочные эффекты со стороны ЖКТ, а именно тошноту и рвоту (табл. 2). Более серьезные побочные эффекты включают панкреатит и медуллярную карциному щитовидной железы, хотя в исследованиях на людях о раке щитовидной железы не сообщалось [1]. Фентермин/топирамат ассоциирован с риском развития тяжелых сердечно-сосудистых осложнений, в то время как прием орлистата увеличивал риск почечной недостаточности и гепатотоксичности [1]. С другой стороны, семаглутид снижал смертность от сердечно-сосудистых заболеваний на 26% по сравнению с плацебо [38]. Семаглутид не влияет на общую функцию почек и считается безопасным для применения у пациентов с умеренной почечной недостаточностью [32].

В Российской Федерации зарегистрированным и рекомендованным к использованию у пациентов с ожирением является сибутрамин [47]. На сегодняшний день отсутствуют исследования, посвященные прямому сравнению эффективности семаглутида с сибутрамином, однако имеются данные сравнения с другими АР ГПП-1, включая лираглутид [48][49]. Было показано, что через 6 мес терапии снижение массы тела >5% отмечено у 91% пациентов группы лираглутида и у 88% группы сибутрамина [49]. Спектр побочных эффектов сибутрамина включает нарушения со стороны ЖКТ (5,1% случаев), сердечно-сосудистой системы (10,5% случаев), нервной системы (5,1% случаев), что обусловлено присущими ему симпатомиметическими свойствами [48][49]. Напротив, семаглутид может оказывать кардиопротекторное действие, что важно для пациентов с ожирением [16].

Основываясь на этих выводах о безопасности, можно отметить, что семаглутид может стать лучшей альтернативой препаратам против ожирения, представленным в настоящее время на рынке. Следует также отметить, что в качестве инкретиновой терапии семаглутид (как и другие АР ГПП-1) имеет низкий риск развития гипогликемии.

## Побочные эффекты и противопоказания

Как мы описывали ранее, наиболее частыми побочными эффектами семаглутида во время клинических исследований SUSTAIN были проявления со стороны ЖКТ, включая тошноту и рвоту [1]. Пациенты, принимавшие семаглутид, испытывали эти побочные эффекты чаще, чем пациенты из групп препаратов сравнения (в исследованиях SUSTAIN, PIONEER и STEP), но большинство эпизодов были преходящими [4]. Повышенная частота возникновения тошноты и рвоты наблюдалась при более высоких дозах семаглутида и более низком исходном ИМТ [4]. Существуют также опасения по поводу повышения уровня панкреатической липазы и частоты развития панкреатита, аналогичных другим АР ГПП-1. Статистически значимое повышение уровня липазы было обнаружено как в ходе исследований SUSTAIN, так и в ходе исследований PIONEER [20][31][50]. У одного пациента в исследовании SUSTAIN 5 развилась метастатическая карцинома поджелудочной железы примерно через 65 дней после начала лечения [20]. Терапия АР ГПП-1 противопоказана пациентам с хроническим или идиопатическим острым панкреатитом в анамнезе [21].

Сообщалось о высокой частоте возникновения ретинопатии, включая кровоизлияние в стекловидное тело и слепоту, у пациентов, получавших семаглутид, по сравнению с плацебо [16]. Считалось, что это связано с быстрым снижением гликемии, а не с прямым эффектом семаглутида. Побочные эффекты, связанные с желчным пузырем, включая желчнокаменную болезнь, варьировались от 0,2 до 4,9%, а сердечно-сосудистые, включая тахикардию и аритмии, – от 1,5 до 9,8% в исследованиях STEP 1–4. Противопоказания к приему семаглутида включают наличие в семейном или личном анамнезе множественной эндокринной неоплазии 2-го типа, нарушение функции почек или медуллярный рак щитовидной железы. Эти ограничения основаны на результатах исследований на животных моделях [1][21].

## Ограничения настоящего исследования

Настоящий обзор литературы имеет несколько ограничений. Три исследования программы SUSTAIN — многорегиональное клиническое исследование SUSTAIN China, SUSTAIN (Japan) и SUSTAIN (Japan, ситаглиптин) — были исключены из-за избыточности сравниваемых препаратов [17–19]. Обзор также включает поверхностные сравнения результатов исследований SUSTAIN, PIONEER и STEP, поскольку до настоящего времени ни в одном исследовании не сравнивалась эффективность подкожного и перорального семаглутида. Эти сравнения нельзя считать окончательными выводами из-за их косвенного характера. Будущие исследования, направленные на сравнение этих двух препаратов, стали бы полезным дополнением к литературе об эффективности семаглутида.

## ЗАКЛЮЧЕНИЕ

Семаглутид — АР ГПП-1, недавно одобренный FDA для контроля веса в дозе 2,4 мг 1 раз в неделю у пациентов с ИМТ ≥30 кг/м2 или ≥27 кг/м2 с более чем одним сопутствующим заболеванием, связанным с избыточным весом. Семаглутид обладает высокой эффективностью, и на сегодняшний день у большинства пациентов наблюдается клинически значимое снижение веса в ходе поэтапных исследований SUSTAIN, PIONEER и STEP. Его эффективность и безопасность были продемонстрированы в течение 2 лет, что делает его идеальным вариантом для долгосрочной терапии ожирения, однако применение может быть ограничено высокой стоимостью. Анализ экономической эффективности поможет клиницистам решить, следует ли отдавать ему предпочтение по сравнению с другими препаратами для снижения массы тела.

## ДОПОЛНИТЕЛЬНАЯ ИНФОРМАЦИЯ

Источники финансирования. Работа выполнена по инициативе авторов без привлечения финансирования.

Конфликт интересов. Авторы декларируют отсутствие явных и потенциальных конфликтов интересов, связанных с содержанием настоящей статьи.

Участие авторов. Шабутдинова О.Р. — разработка концепции и дизайна исследования, получение и анализ данных, интерпретация результатов; Даутов А.Р. — разработка дизайна исследования, написание статьи; Самков А.А. — анализ данных, написание статьи; Кононенко А.В. — концепция исследования; Саргалиев А.Ф. — получение и анализ данных, редактирование статьи; Давлетшин А.Р. — интерпретация результатов, редактирование статьи; Андресова П.А. — анализ данных, редактирование статьи; Зарбеева К.Р. — получение данных, редактирование статьи; Торшхоева Д.А. — интерпретация результатов; Рахмонкулов У.А. — написание и редактирование статьи; Афанасьев А.А. — написание и редактирование статьи. Все авторы внесли равный вклад в написание статьи и одобрили ее финальную версию перед публикацией, выразили согласие нести ответственность за все аспекты работы, подразумевающую надлежащее изучение и решение вопросов, связанных с точностью или добросовестностью любой части работы.
